# Enhancing genomics-based outbreak detection of endemic *Salmonella enterica* serovar Typhimurium using dynamic thresholds

**DOI:** 10.1099/mgen.0.000310

**Published:** 2019-11-04

**Authors:** Michael Payne, Sophie Octavia, Laurence Don Wai Luu, Cristina Sotomayor-Castillo, Qinning Wang, Alfred Chin Yen Tay, Vitali Sintchenko, Mark M. Tanaka, Ruiting Lan

**Affiliations:** ^1^​ School of Biotechnology and Biomolecular Sciences, University of New South Wales, Sydney, New South Wales, Australia; ^2^​ Centre for Infectious Diseases and Microbiology – Public Health, Institute of Clinical Pathology and Medical Research, Westmead Hospital, New South Wales, Australia; ^3^​ Marie Bashir Institute for Infectious Diseases and Biosecurity, University of Sydney, Westmead NSW, New South Wales, Australia; ^4^​ Pathology and Laboratory Medicine, University of Western Australia, Perth, Western Australia, Australia

**Keywords:** genomic epidemiology, outbreak detection, *Salmonella *Typhimurium, bacterial population genomics, genetic clustering

## Abstract

*
Salmonella enterica* serovar Typhimurium is the leading cause of salmonellosis in Australia, and the ability to identify outbreaks and their sources is vital to public health. Here, we examined the utility of whole-genome sequencing (WGS), including complete genome sequencing with Oxford Nanopore technologies, in examining 105 isolates from an endemic multi-locus variable number tandem repeat analysis (MLVA) type over 5 years. The MLVA type was very homogeneous, with 90 % of the isolates falling into groups with a five SNP cut-off. We developed a new two-step approach for outbreak detection using WGS. The first clustering at a zero single nucleotide polymorphism (SNP) cut-off was used to detect outbreak clusters that each occurred within a 4 week window and then a second clustering with dynamically increased SNP cut-offs were used to generate outbreak investigation clusters capable of identifying all outbreak cases. This approach offered optimal specificity and sensitivity for outbreak detection and investigation, in particular of those caused by endemic MLVA types or clones with low genetic diversity. We further showed that inclusion of complete genome sequences detected no additional mutational events for genomic outbreak surveillance. Phylogenetic analysis found that the MLVA type was likely to have been derived recently from a single source that persisted over 5 years, and seeded numerous sporadic infections and outbreaks. Our findings suggest that SNP cut-offs for outbreak cluster detection and public-health surveillance should be based on the local diversity of the relevant strains over time. These findings have general applicability to outbreak detection of bacterial pathogens.

## Data Summary

Sequencing data have been deposited at the National Center for Biotechnology Information under BioProject number PRJNA555108.

**Impact Statement**

Whole-genome sequencing (WGS) offers the ultimate resolution for strain typing in outbreak investigations. However, the question of how to rule in or rule out an isolate of an outbreak remains largely unsolved. Variation in genetic diversity of the local population of a given pathogen means that genetic distance cut-offs need to be adjusted accordingly. In this study, the genetic diversity of 105 isolates of *Salmonella enterica* serovar Typhimurium within one MLVA (multi-locus variable number tandem repeat analysis) type isolated over 5 years, including isolates from three epidemiologically confirmed outbreaks, was examined using WGS. We describe a two-stage clustering method that includes an initial zero distance cut-off, followed by a dynamic cut-off which is calculated using both genetic and temporal distances. This method successfully identified all three epidemiologically defined clusters, as well as identifying five additional clusters that may represent previously undetected outbreaks. These eight clusters were made up of 38 isolates (36 % of the isolates analysed) that would have been deemed outbreak related for investigation. The application of this system optimizes sensitivity and specificity of outbreak detection, and should allow a more efficient public-health response to outbreak control.

## Introduction

*Salmonella enterica* serovar Typhimurium is the most common serovar causing salmonellosis in Australia. In the state of New South Wales (NSW), multi-locus variable number tandem repeat analysis (MLVA) has been routinely used for subtyping and public-health laboratory surveillance since 2006. Epidemiological investigations are triggered when there are at least five cases of *S *. *enterica* Typhimurium infections with the same MLVA type within a 4 week window period. However, common or endemic MLVA types may cause multiple outbreaks along with sporadic cases and a few prevalent MLVA types may represent a large portion of isolates observed in a geographical location, leading to a reduced effectiveness of MLVA to detect outbreaks [1].

Whole-genome sequencing (WGS) has increasingly been implemented in public-health laboratories for epidemiological typing and outbreak investigation [2–4]. Previously, we applied WGS to retrospectively examine 57 *S. enterica* Typhimurium isolates from five epidemiologically confirmed outbreaks [5]. These outbreaks were initially detected by MLVA typing and have been previously investigated by NSW Health Protection. We found that most of the isolates confirmed epidemiologically to be involved in the outbreaks were either identical or differed by one to two SNPs, if the food source of the outbreak was contaminated by only a single strain. In addition, WGS analysis ruled in isolates that were initially not considered to be linked with the outbreak. This additional insight increased the total outbreak size by 107 %.

The selection of the SNP difference cut-off for isolates to be considered as the same outbreak remains a major challenge for WGS-based surveillance. In our previous study, we modelled the mutation process to derive such a cut-off for ‘ruling-in’ or ‘ruling-out’ a possible epidemiological link. We found that for an outbreak with less than 1 month ‘evolution’ time, the maximum number of SNP differences between isolates was four SNPs using the fastest recorded *Salmonella* mutation rate [5]. However, this threshold was not useful for an endemic MLVA type in which background isolates may differ from outbreak isolates by only one SNP [1]. Our study, as well as many others [1, 5–8], have shown the added value of WGS in the investigation of point-source community outbreaks of *S *. *enterica* Typhimurium. However, many studies examined sets of isolates from confirmed outbreaks and fewer examined larger populations to place these outbreaks in a broader context [9–11]. A large-scale retrospective analysis from random samples of an endemic group of *S *. *enterica* Typhimurium over several years could offer further insight into the effect of genomic variability on defining outbreaks. Therefore, in this study, we explored the utility of second (Illumina) and third (Oxford Nanopore) generation sequencing to distinguish *S *. *enterica* Typhimurium outbreaks from very closely related isolates of the same endemic MLVA type using selected isolates from 2010 to 2014. We examined the need to incorporate local genetic diversity as well as temporal data when determining SNP thresholds for outbreak detection, and evaluated the usefulness of complete genome assembly for outbreak detection. We also characterized the evolution and shared characteristics of an endemic MLVA type.

## Methods

### Strains and genome sequencing

A total of 105 *S *. *enterica* Typhimurium DT170 isolates with MLVA type 3-9-7-12-523 isolated in NSW from 2010 to 2014 were sequenced using a paired-end library on a MiSeq (Illumina) platform. Metadata for these isolates were used with ethics approval. Eighteen of these strains were sequenced using long-read sequencing with Oxford Nanopore technologies. Library preparation was performed with either a ligation sequencing kit 1D (SQK-LSK108) and native barcoding kit (EXP-NBD103) or a rapid barcoding kit (SQK-RBK004). For the native barcoded library, the library was prepared as described by Wick *et al*. [12], while the rapid barcoded library was prepared according to the manufacturer’s instructions. The barcoded libraries were then loaded onto an R9.4.1 flow cell (FLO-MIN106) and sequenced on the MinION MK1b for 48 h. Local base calling and demultiplexing of fast5 reads was performed using Albacore v2.3.3 (Oxford Nanopore). Read statistics were generated using NanoStat v1.1.2 [13]. Hybrid assembly was performed with Unicycler v0.4.7 [14] using the long-read assembly produced from Canu v1.7 [15]. The genome sequencing data (Illumina and Oxford Nanopore) are available from the National Center for Biotechnology Information (NCBI) under BioProject number PRJNA555108.

### SNP calling and phylogeny generation

SNPs were determined using snippy [16] with ‘--mapqual 30’, ‘--mincov 15’ and ‘--minfrac 0.9’ settings using the LT2 reference (NC_003197.2). Snippy includes SnpEff, which provided functional consequence predictions for each of the SNPs [17]. Non-synonymous SNP effect prediction was performed with Provean using default settings [18]. Nucleotides for each position where a SNP was called were extracted for each strain using a custom Python script to generate an alignment (supplementary_data_snp_align_gen.py). This included the reference sequence for isolates without a SNP and also ‘N’ characters where the position could not be accurately called. SNPs located on phage regions defined by phaster using default settings [19], insertion sequence (IS) elements or CRISPRs were excluded. SNP locations that were called ‘N’ in greater than 20 % of isolates were removed. The phylogeny was constructed using mega 7 [20] with the maximum parsimony algorithm, using the tree bisection and reconnection (TBR) swap method. The tree and associated metadata were visualized using iTOL [21]. SNP clusters were identified by single linkage clustering with maximum genetic distance cut-offs of zero to five SNPs. In this context, single linkage clustering means the grouping of all isolates connected by the same or fewer differences than a defined cut-off. Structural variation in completely sequenced genomes was identified using progressiveMauve [22] alignments and confirmed by examination of read mapping in igv [23]. phaster was used with default settings to identify the phage complement of complete genomes [19].

## Results

### Selection, sequencing and phylogenomic analysis of 105 MLVA type 3-9-7-12-523 isolates

We selected 105 *S. enterica* Typhimurium DT170 isolates for sequencing and further analysis (Table S1, available with the online version of this article) as they were geographically and temporally distributed. These isolates were selected out of 615 in total over the 5 year period, with 14 to 27 isolates per year, including isolates from three known outbreaks. The isolates shared MLVA type 3-9-7-12-523, which was an endemic type in the state of NSW, Australia [24], where it was amongst the most frequently identified MLVA types over a 7 year period (Table 1). The genomes were sequenced using Illumina sequencing with a mean of 45-fold coverage. A total of 817 SNPs was found relative to the LT2 reference, with 545 of these common to all strains and 272 only found amongst isolates within the MLVA type (Table S2). Small indels were not examined.

**Table 1.** Frequency of the 3-9-7-12-523 MLVA type in NSW, Australia, from 2007 to 2014.

Year	No. of 3-9-7-12-523 isolates	Total no. of isolates	Percentage	Rank*
2007	3	247	1.21	11 (115)
2008	28	1240	2.26	9 (233)
2009	211	1604	13.15	2 (292)
2010	36	973	3.70	3 (247)
2011	292	2104	13.87	1 (375)
2012	116	1677	6.91	3 (293)
2013	55	1980	2.77	9 (379)
2014	116	2287	5.07	4 (322)

*Numbers in brackets are the total number of MLVA types in that year.

**Fig. 1.**
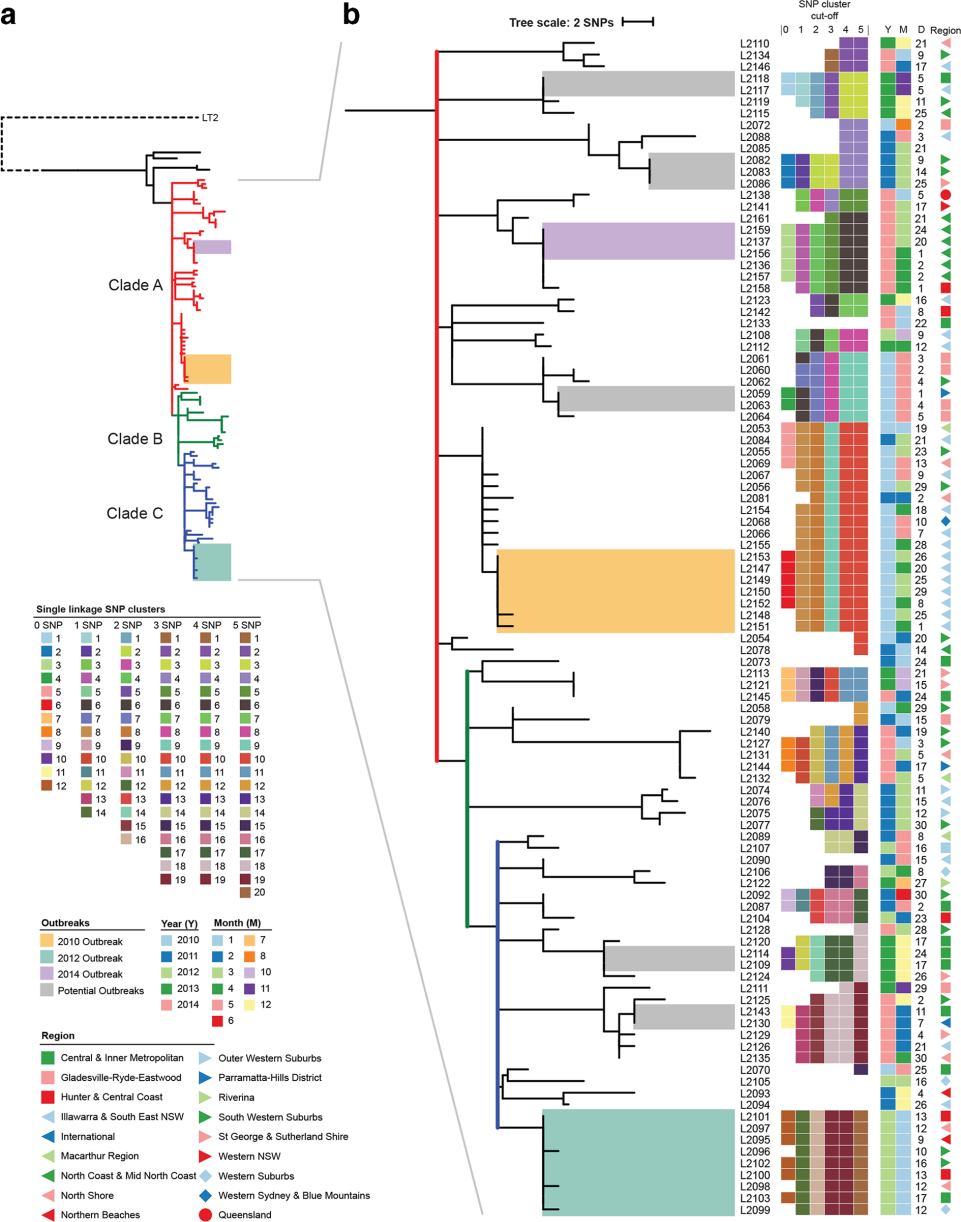
Maximum parsimony phylogeny of 105 *S *. *enterica* Typhimurium isolates from MLVA type 3-9-7-12-523 from NSW from 2010 to 2014. (a) An overview of the phylogeny, which includes the three major clades (A in red, B in green and C in blue) that include 101 of 105 isolates in the MLVA type. The remaining isolates are shown in black, as is the LT2 reference that is used as an outgroup. (b) A detailed view of the three large clades including the three known outbreaks from 2010, 2012 and 2014. Ancestral nodes for each of clade A, B and C are highlighted in red, green and blue, and have eight, four and seven branches, respectively. All of these branches arise from a polytomy in each case, highlighting the low genetic diversity of the source strain. The year (**Y**), month (**M**), day (**D**) and region of isolation for each isolate are shown, as are single linkage SNP clusters with zero to five SNP cut-offs. At each cut-off, isolates are grouped into clusters, and each cluster is assigned a cluster number and corresponding colour.

A phylogeny based on the SNPs identified was generated to examine the relationships of isolates within the MLVA group (Fig. 1). All but four isolates clustered together in one clade (clade A) and the maximum pairwise distance within this group was only 32 SNPs. Interestingly, the common ancestor of clade A (marked in red in Fig. 1) is located at a polytomy that gives rise to eight distinct subclades and the maximum divergence of any of its descendants is 18 SNPs. Similarly, the largest of these eight subclades (clade B) also contains a polytomy (marked in green) with four subclades arising from it. Finally, one of these four subclades (clade C) is again a polytomy (marked in blue) with six subclades. While these polytomies may be due to under-sampling, the very low overall diversity suggests that the majority of isolates from MLVA type 3-9-7-12-523 were derived from a single and recent common ancestor that has infected humans on numerous independent occasions.

### SNP threshold for outbreak cluster detection within the MLVA type

**Fig. 2.**
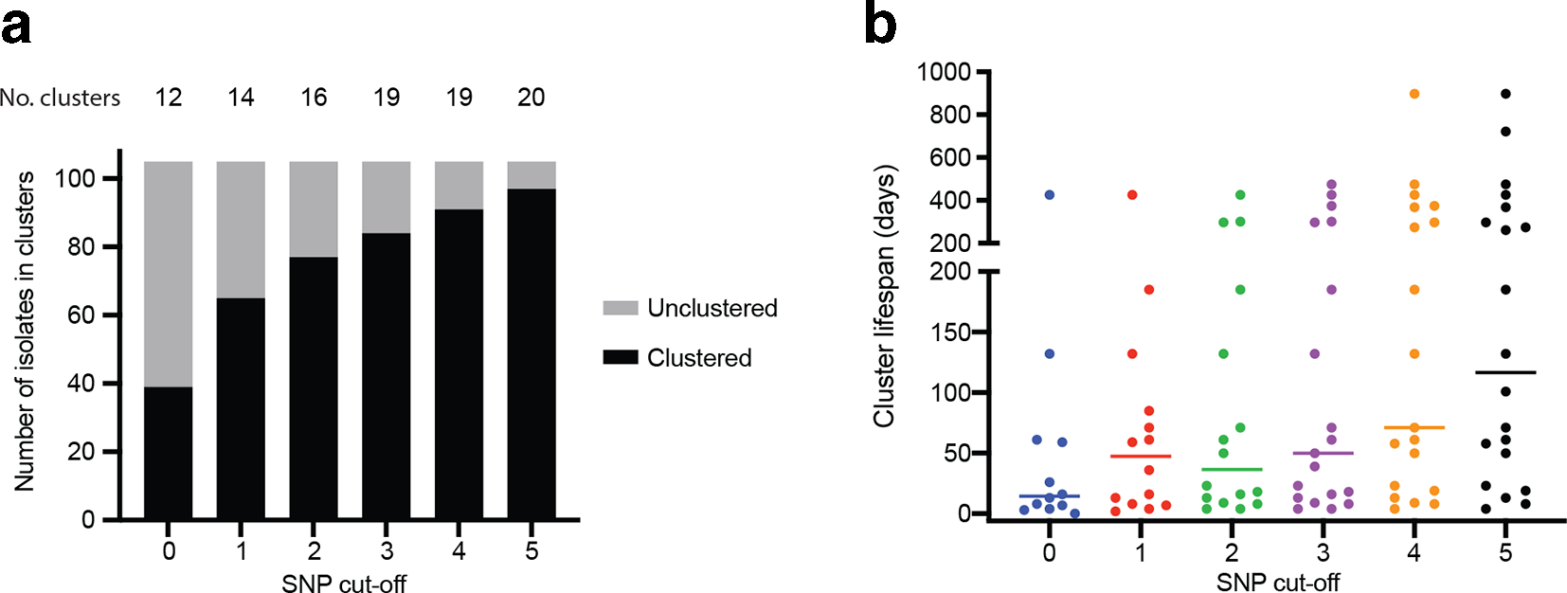
Characteristics of zero to five SNP clusters derived from 105 MLVA type 3-9-7-12-523 isolates. (a) The number of isolates that are grouped into SNP clusters by single linkage clustering at each of six cut-offs from zero to five SNPs. The number of clusters at each level is shown above each column. (b) The period of time between isolation of the first isolate and the last (lifespan) for each cluster in days at each SNP cut-off. Median cluster length is shown with horizontal bars.

In order to further characterize the isolates, single linkage clusters of two or more isolates were identified with either zero, one, two, three, four or five SNP differences. We used a minimum cluster size of two as we only sampled 17 % of the total number of isolates of the given MLVA type. The number of clusters increased with the increase in allowable differences as expected (Fig. 2a). The percentage of isolates falling into a cluster varied from 37 % at the zero SNP cut-off to 92 % at the five SNP cut-off. We also examined the lifespan of the clusters. For zero SNP clusters, only 1 of the 12 clusters spanned a period of over 1 year with a median lifespan of 14.5 days, whereas for the five SNP cluster the longest lifespan was 898 days with a median of 116.5 days (Fig. 2b). Therefore, the larger the SNP cut-off, the higher the likelihood of the inclusion of temporally distant isolates that are unlikely to be part of an outbreak.

We further classified clusters as either existing within one 4 week period or spanning more than 4 weeks. A 4 week window was employed as it was the default period for outbreak cluster detection using MLVA in Australia [24]. Three epidemiologically defined outbreaks that occurred in 2010, 2012 and 2014 contained 7, 9 and 3 isolates, respectively (Fig. 1). At the zero SNP cut-off, all three outbreaks were detected and fell within a 4 week window. However, two of the seven isolates in the 2010 outbreak and three of the nine isolates in the 2012 outbreak differed by one SNP and would be excluded from the outbreak. At the one SNP cut-off, there were an additional ten isolates spanning 5 months included in the outbreak cluster for the 2010 outbreak and three additional isolates spanning 2 months falling into the 2014 outbreak cluster. Therefore, a threshold of zero SNPs as a cut-off would be more appropriate than one SNP difference for detection of point-source outbreaks of a short duration for this MLVA type.

Five 0 SNP clusters (clusters 1, 2, 4, 10 and 11) were each contained within separate 4 week windows, but were not previously investigated. Clusters 2, 4 and 10 all arose exclusively from locations in metropolitan Sydney, and were found within 16, 3 and 7 day periods. Both isolates from cluster 1 were from the same day but were identified in different regions of the state, while cluster 11 included one isolate from metropolitan Sydney and one from an overseas traveller separated by 4 days. The spatial and temporal data suggest that these were likely outbreak clusters.

### Different SNP number thresholds and trade-off between sensitivity and specificity of cluster detection

As described above, zero SNP clusters had high specificity because they only identified isolates within the outbreak when considering the three confirmed outbreak clusters. However, this stringent cut-off came at a cost of sensitivity where other isolates that were part of these outbreaks were excluded. One solution to this problem is to define a second dynamic SNP cut-off to determine the level most likely to contain all epidemiologically linked isolates, i.e. to identify the ‘investigation cluster’ to include all likely cases for outbreak investigation. Starting with each zero SNP cluster, the cut-off is increased until the cluster lifespan is more than 4 weeks, i.e. contained isolates from outside the defined time period. The largest SNP cut-off that restricts the cluster to a 4 week window is then used to identify cases for epidemiological investigation. This broader cluster is then named as the outbreak investigation cluster as opposed to the outbreak detection cluster, which provides an initial trigger for outbreak investigation.

Using this approach, the 2012 outbreak zero SNP detection cluster can be increased to a five SNP investigation cluster while maintaining a 4 week lifespan, resulting in three additional outbreak isolates being included (Table S1). The 2014 outbreak can also be expanded to five SNPs to include four additional isolates. By contrast, the 2010 outbreak can only be defined using the zero SNP cut-off, meaning that the detection and investigation clusters are the same. The approach was also applied to previously uncharacterized clusters. The zero SNP detection cluster 4 can be expanded to a five SNP investigation cluster to include four additional isolates, while zero SNP detection cluster 10 can be expanded to a four SNP investigation cluster with two additional isolates. Both of these larger clusters are found within the same or neighbouring geographical regions, suggesting that in these cases the inclusion of isolates from more genetically diverse clusters may increase the sensitivity of detection of potential outbreak cases.

### Utility of complete sequencing in genomic surveillance

To further investigate the epidemiologically defined outbreaks and to evaluate the usefulness of additional Nanopore sequencing for outbreak investigation, complete genomes from 18 isolates were generated with hybrid assembly using Nanopore and Illumina reads. We investigated cases where there were no or very few SNP differences between isolates that might have been expected to be more genetically distinct. These included five isolates (L2159, L2137, L2156, L2136 and L2157) that were indistinguishable from each other using SNP analysis despite the last two isolates not being assigned to the outbreak that the first three isolates were assigned to. We also included isolates from the 2010 outbreak cluster where the outbreak isolates L2153, L2147, L2149, L2150 and L2152 were separated by one SNP from L2084, a non-outbreak isolate obtained almost 12 months later. Genomic changes such as phage and transposon movement, larger insertions/deletions and genomic rearrangements can only be accurately identified using complete genome assembly. However, after analysing all of the above-mentioned isolates using additional Nanopore sequencing, none of these variations were identified and so no additional insight was obtained.

While no additional changes were identified within the MLVA type, two large deletions were identified in all 18 isolates relative to *S. enterica* Typhimurium strain LT2. The first was 498 bp in length and found in STM0291, which encodes a component of a type 6 secretion system found in *Salmonella* pathogenicity island 6 (SPI6). Interestingly, this deletion does not cause a frameshift and results in the deletion of three RHS (rearrangement hot spot) repeats. The second is a 195 bp in-frame deletion in the variable PQ repeat region found in the *ftsK* gene, which functions in cell division. This protein is considered essential; however, its gene length varies between serovars [25] and a VNTR (variable number tandem repeat, named STTR7) is located within the gene, which is used in MLVA schemes in several serovars but not in *S. enterica* Typhimurium [26, 27].

Deletions were also identified in the pSLT plasmid. The L2084 isolate had a 119 bp deletion in the *spvA* gene, part of the *spv* virulence locus that promotes intracellular growth during invasive infection [28]. The L2159 isolate contained a 42 bp deletion in *repA2,* which encodes a protein involved in plasmid replication. All 18 isolates contained an 86 bp deletion in the *traD* gene, which is required for conjugation and transfer of pSLT. This deletion has been identified previously and is known to lead to a loss of function in the encoded protein [29].

All 18 isolates that were completely sequenced by Nanopore contain prophage sequences consistent with an isolate of the same MLVA type in a previous study [30]. All contained Gifsy-1, Gifsy-2 (DT2 type), P4, SPN9CC and ST64B prophages. ISs that were either gained or lost relative to LT2 were also examined. Two ISs (tnp_A4 – IS*200* and tnp_A6 – IS*200*) were lost in all 18 isolates, while 4 additional IS*200*F family transposons were inserted in all 18 isolates. Three of these IS element copies are located in intergenic regions while the fourth is located within the ORF of STM1228, which encodes a putative periplasmic protein of unknown function.

## Discussion

In this study, we examined genome sequences of 105 isolates of an endemic MLVA type from a 5 year period to determine the genomic variation of the isolates and assess SNP cut-offs for outbreak detection and public-health surveillance. The 3-9-7-12-523 MLVA type isolates were very homogeneous, with 90 % falling into groups with a five SNP cut-off. Many of these clusters were derived from polytomies and, therefore, were derived from strains of the same source. The best cut-off for outbreak detection was zero SNPs as it contained the fewest SNP clusters isolated over periods greater than 4 weeks. We further developed a dynamic cut-off for outbreak investigation based on these zero SNP clusters. Complete genomes were generated using Oxford Nanopore to allow the complete examination of additional genetic variation between isolates within the MLVA type, but no such additional variation was detected.

### Determining SNP cut-off for outbreak detection requires knowledge of local diversity of the isolates over time

There have been several attempts to derive empirical genome variability cut-offs for outbreak cluster detection [7, 31, 32]. Clearly, no single cut-offs can be derived, with recommendations ranging from 0 to over 10 SNPs [7, 31, 32]. We modelled the SNP cut-offs previously based on *S. enterica* Typhimurium mutation rate and isolate evolution time, and found four SNPs offered an optimal threshold of sensitivity to detect outbreaks and specificity to exclude non-outbreak isolates [5]. However, in our previous case study of an endemic MLVA type, a four SNP cut-off was still too lax as background isolates differed from outbreak isolates by only one SNP [1]. For the MLVA type in this study, a cut-off of zero SNPs offered the best specificity for outbreak detection with 67 % of SNP clusters (8/12) found within a 4 week window, including all three epidemiologically defined outbreaks. Our results showed that isolates with zero or one SNP differences can be recovered over several years, suggesting a low mutation rate of *S. enterica* Typhimurium genomes in the original source or potentially inflated estimates of mutation rate in previous studies. Therefore, determining an appropriate SNP cut-off for outbreak cluster detection requires an understanding of the local diversity of the isolates over time. Also, the two stage clustering process is reliant on the presence of closely related non-outbreak isolates such as those found within the endemic MLVA type. Therefore, we suggest retrospective sequencing of isolates from previous years with at least 1 year of data will be necessary for the initial set up of WGS for outbreak detection in routine public-health applications.

Five of the eight 0 SNP clusters within a 4 week window identified in this study were found in close geographical proximity, suggesting that these were potential outbreak clusters. However, the other three clusters of isolates were found across large distances. The addition of spatial data provided further support to temporal and genetic data for potential outbreak clusters. However, spatial data may not be entirely reliable given the ease of long-distance travel of both food and people.

### Epidemiological investigation may use a second, dynamic SNP cut-off to increase the sensitivity of outbreak case detection

The use of the zero SNP cut-off can affect the sensitivity of the technique because isolates with one or more SNP differences potentially from the same outbreak could be excluded despite being from the same location and in some cases the same day of the disease onset. To increase sensitivity, we suggest that initial outbreak detection clusters of zero SNPs should be complemented with a second, larger cut-off for outbreak investigation to include likely cases. The cut-off for the investigation cluster is dynamic and derived separately for each outbreak detection cluster. The use of this two-stage clustering for outbreak detection and investigation maintains specificity while increasing sensitivity. It avoids triggering investigation of false outbreaks or underestimating the size of a genuine outbreak. For the data we analysed in this study, the number of cases that would need to be investigated would be reduced from 97 (at a static five SNP cut-off) to 38 (utilizing dynamic investigation cluster cut-offs from zero to five SNPs). Further work is required to validate this approach on more recent, larger datasets.

The effectiveness of the two-stage clustering depends on the diversity of related background isolates as well as a 4 week window, which is based on current epidemiological investigation practice using MLVA [33]. This temporal window may be shortened for WGS-guided investigations due to the increased sensitivity of the technique. However, further study is required to identify the optimal window size and minimum number of cases for sensitivity and specificity of outbreak detection.

There may be situations where a single outbreak is caused by more than one strain. In this scenario, two initial detection clusters may be identified. These clusters may or may not merge to become one investigation cluster depending on the genetic distance between the two strains. Regardless, epidemiological data could be used to trace both clusters to the same source, which would then suggest a multi-strain outbreak. In a multi-strain outbreak, it is possible that one strain may cause fewer cases and may not be included in detection/investigation clusters, these scenarios can occur and cannot be resolved by WGS alone. However, this situation is not typical of single point source outbreak scenarios we have previously examined [1, 5].

As is the case with any SNP-based approach, the choice of reference and variant caller as well as the quality of input data may impact results. Ideally, the closest possible complete reference genome should be used when calling SNPs as a distant reference can lead to the omission of some SNPs [25]. However, in practice it is not feasible to use an internal complete reference from an outbreak in a public-health investigation setting, because the generation of a complete genome requires additional sequencing and analysis. The potential under-detection of SNPs may lead to false outbreak detection cluster formation (false-positive clusters) and may also have an effect on outbreak investigation cluster size (false-positive cases). However, we expect there will be very few undetected SNPs, which will have minimal impact on the outcomes of outbreak detection. Additionally, due to the high sensitivity of the approach (zero SNP clusters) small differences between variant callers or poor-quality input data could lead to inconsistent results. These issues should be mitigated through the development and implementation of standardized, validated SNP calling pipelines.

### Illumina sequencing is sufficient for accurate outbreak detection

We utilized Illumina and Nanopore sequencing based hybrid assembly in an attempt to identify any further genomic changes that could distinguish isolates within clusters that were epidemiologically unlinked to outbreaks. The combination of Illumina and Nanopore data is necessary for SNP detection because Nanopore data alone are not as accurate at the nucleotide level [34]. Additional mutations common to all of the isolates were identified, including large insertions/deletions, prophage complement changes and IS insertions. However, no additional mutations specific to subsets of isolates within the MLVA group were detected. The contrast between 562 SNPs and zero rearrangements relative to the reference strain LT2 suggests that the genome rearrangement rate is several hundred times lower than the SNP mutation rate and, thus, these large genomic changes offer little improvement of resolution for outbreak investigations.

### Low genetic diversity of the MLVA type points to the existence of a persistent reservoir causing ongoing infections

The variation seen within the MLVA type shows that all but four isolates were found within a single 12 SNP single linkage cluster and shared a common ancestor relatively recently. This large group is characterized by three clades,the original ancestral type (clade A),a clade derived from it (clade B) and then a subsequent clade (clade C) derived from clade B. Isolates from later dates were not predominantly derived from the more diverged B and C subclades. Instead,both the original source clade and the two derived clades continued to seed human infection over the 5 years of the study. This fact and the large proportion of polytomies at the root of 0–5 SNP clusters suggests that the MLVA type was likely present as a pool of closely related source isolates that continued to cause multiple separate human infection clusters over time. A similar group of outbreaks was recently traced back to an egg grading facility [11]. These outbreaks occurred over a shorter time span. However,they showed a similar phylogenetic structure of multiple polytomies. Given the potential of source tracing to farm level and the power of WGS,a concerted effort of public health and food safety will ultimately identify and eliminate the organism at its primary source. The MLVA type examined here has persisted for 5 years and multiple SNP clusters of less than five SNP differences have persisted for months,giving ample opportunity to perform primary source tracing to reduce the burden of foodborne endemic *S *. *enterica* Typhimurium infections.

### Conclusion

This study investigated the genomic variation found within one of the most commonly identified and endemic MLVA types in Australia, and examined how genetic distance cut-offs can be set for the reliable and specific identification of point-source outbreak isolates. A new outbreak detection approach using WGS and a dynamic, two-step single linkage clustering process appears to offer improved specificity and sensitivity for outbreak detection and case inclusion for subsequent investigation by public-health authorities. The initial identification of a highly specific outbreak detection cluster followed by expansion of these clusters into dynamic outbreak investigation clusters provides a means to limit epidemiological investigations to only the most relevant cases. This new approach will be applicable to outbreak investigation of other bacterial pathogens and will be particularly useful in endemic MLVA types, clones or species with low genetic diversity. The use of a dynamic cut-off will reduce the need to examine phylogenetic trees to delineate outbreak clusters, making the process more amenable to automation for rapid public-health applications. The complete genome assembly using Nanopore data did not provide additional resolution and Illumina sequencing is likely sufficient for outbreak detection for *S *. *enterica* Typhimurium. Finally, the phylogenetic relationships within this MLVA type suggests that all of the isolates from this endemic type were derived from a single source that repeatedly caused human infections.

## Data bibliography

Payne M, Octavia S, Luu LDW, Sotomayor-Castillo C, Wang Q, Tay ACY, Sintchenko V, Tanaka MM, Lan R. All the sequencing data used in this study were generated within the study, NCBI BioProject number PRJNA555108 (2019).

## Supplementary Data

Supplementary material 1

Supplementary material 2

## References

[1] Phillips A, Sotomayor C, Wang Q, Holmes N, Furlong C (2016). Whole genome sequencing of *Salmonella* Typhimurium illuminates distinct outbreaks caused by an endemic multi-locus variable number tandem repeat analysis type in Australia, 2014. BMC Microbiol.

[2] Ford L, Carter GP, Wang Q, Seemann T, Sintchenko V (2018). Incorporating whole-genome sequencing into public health surveillance: lessons from prospective sequencing of *Salmonella* Typhimurium in Australia. Foodborne Pathog Dis.

[3] Mook P, Gardiner D, Verlander NQ, McCormick J, Usdin M (2018). Operational burden of implementing *Salmonella* Enteritidis and Typhimurium cluster detection using whole genome sequencing surveillance data in England: a retrospective assessment. Epidemiol Infect.

[4] Anonymous (2018).

[5] Octavia S, Wang Q, Tanaka MM, Kaur S, Sintchenko V (2015). Delineating community outbreaks of *Salmonella enterica* serovar Typhimurium by use of whole-genome sequencing: insights into genomic variability within an outbreak. J Clin Microbiol.

[6] Mair-Jenkins J, Borges-Stewart R, Harbour C, Cox-Rogers J, Dallman T (2017). Investigation using whole genome sequencing of a prolonged restaurant outbreak of *Salmonella* Typhimurium linked to the building drainage system, England, February 2015 to March 2016. Euro Surveill.

[7] Gymoese P, Sørensen G, Litrup E, Olsen JE, Nielsen EM (2017). Investigation of outbreaks of *Salmonella enterica* serovar Typhimurium and its monophasic variants using whole-genome sequencing, Denmark. Emerg Infect Dis.

[8] Bloomfield SJ, Benschop J, Biggs PJ, Marshall JC, Hayman DTS (2017). Genomic analysis of *Salmonella enterica* serovar Typhimurium DT160 associated with a 14-year outbreak, New Zealand, 1998-2012. Emerg Infect Dis.

[9] Mather AE, Reid SWJ, Maskell DJ, Parkhill J, Fookes MC (2013). Distinguishable epidemics of multidrug-resistant *Salmonella* Typhimurium DT104 in different hosts. Science.

[10] Zhang S, Li S, Gu W, den Bakker H, Boxrud D (2019). Zoonotic source attribution of *Salmonella enterica* serotype Typhimurium using genomic surveillance data, United States. Emerg Infect Dis.

[11] Ford L, Wang Q, Stafford R, Ressler K-A, Norton S (2018). Seven *Salmonella* Typhimurium outbreaks in Australia linked by trace-back and whole genome sequencing. Foodborne Pathog Dis.

[12] Wick RR, Judd LM, Gorrie CL, Holt KE (2017). Completing bacterial genome assemblies with multiplex MinION sequencing. Microb Genom.

[13] De Coster W, D'Hert S, Schultz DT, Cruts M, Van Broeckhoven C (2018). NanoPack: visualizing and processing long-read sequencing data. Bioinformatics.

[14] Wick RR, Judd LM, Gorrie CL, Holt KE (2017). Unicycler: resolving bacterial genome assemblies from short and long sequencing reads. PLoS Comput Biol.

[15] Koren S, Walenz BP, Berlin K, Miller JR, Bergman NH (2017). Canu: scalable and accurate long-read assembly via adaptive *k*-mer weighting and repeat separation. Genome Res.

[16] Seemann T (2015). snippy: fast bacterial variant calling from NGS reads, v3.1. GitHub.

[17] Cingolani P, Platts A, Wang LL, Coon M, Nguyen T (2012). A program for annotating and predicting the effects of single nucleotide polymorphisms, SnpEff: SNPs in the genome of Drosophila melanogaster strain w(1118); iso-2; iso-3. Fly.

[18] Choi Y, Chan AP (2015). PROVEAN web server: a tool to predict the functional effect of amino acid substitutions and indels. Bioinformatics.

[19] Arndt D, Grant JR, Marcu A, Sajed T, Pon A (2016). PHASTER: a better, faster version of the PHAST phage search tool. Nucleic Acids Res.

[20] Kumar S, Stecher G, Tamura K (2016). MEGA7: molecular evolutionary genetics analysis version 7.0 for bigger datasets. Mol Biol Evol.

[21] Letunic I, Bork P (2016). Interactive tree of life (iTOL) V3: an online tool for the display and annotation of phylogenetic and other trees. Nucleic Acids Res.

[22] Darling AE, Mau B, Perna NT (2010). progressiveMauve: multiple genome alignment with gene gain, loss and rearrangement. PLoS One.

[23] Thorvaldsdóttir H, Robinson JT, Mesirov JP (2013). Integrative genomics Viewer (IGV): high-performance genomics data visualization and exploration. Brief Bioinform.

[24] Sintchenko V, Wang Q, Howard P, Ha CW, Kardamanidis K (2012). Improving resolution of public health surveillance for human *Salmonella enterica* serovar Typhimurium infection: 3 years of prospective multiple-locus variable-number tandem-repeat analysis (MLVA). BMC Infect Dis.

[25] Dubarry N, Possoz C, Barre F-X (2010). Multiple regions along the *Escherichia coli* FtsK protein are implicated in cell division. Mol Microbiol.

[26] Kang MS, Kwon YK, Oh JY, Call DR, An BK (2011). Multilocus variable-number tandem-repeat analysis for subtyping *Salmonella enterica* serovar Gallinarum. Avian Pathol.

[27] Vignaud M-L, Cherchame E, Marault M, Chaing E, Le Hello S (2017). MLVA for *Salmonella enterica* subsp. *enterica* serovar Dublin: development of a method suitable for inter-laboratory surveillance and application in the context of a raw milk cheese outbreak in France in 2012. Front Microbiol.

[28] Guiney DG, Fierer J (2011). The role of the spv genes in *Salmonella* pathogenesis. Front Microbiol.

[29] Ahmer BM, Tran M, Heffron F (1999). The virulence plasmid of *Salmonella* Typhimurium is self-transmissible. J Bacteriol.

[30] Fu S, Hiley L, Octavia S, Tanaka MM, Sintchenko V (2017). Comparative genomics of Australian and international isolates of *Salmonella* Typhimurium: correlation of core genome evolution with CRISPR and prophage profiles. Sci Rep.

[31] Bekal S, Berry C, Reimer AR, Van Domselaar G, Beaudry G (2016). Usefulness of high-quality core genome single-nucleotide variant analysis for subtyping the highly clonal and the most prevalent *Salmonella enterica* serovar Heidelberg clone in the context of outbreak investigations. J Clin Microbiol.

[32] Leekitcharoenphon P, Nielsen EM, Kaas RS, Lund O, Aarestrup FM (2014). Evaluation of whole genome sequencing for outbreak detection of *Salmonella enterica*. PLoS One.

[33] Sintchenko V, Wang Q, Howard P, Ha CW, Kardamanidis K (2012). Improving resolution of public health surveillance for human *Salmonella enterica* serovar Typhimurium infection: 3 years of prospective multiple-locus variable-number tandem-repeat analysis (MLVA). BMC Infect Dis.

[34] Tyler AD, Mataseje L, Urfano CJ, Schmidt L, Antonation KS (2018). Evaluation of Oxford Nanopore's MinION sequencing device for microbial whole genome sequencing applications. Sci Rep.

