# Supplementary material for: Enhancing genomics-based outbreak detection of endemic Salmonella enterica serovar Typhimurium using dynamic thresholds
Source: Microb Genom. 2019 Nov 4;7(6):000310. doi: 10.1099/mgen.0.000310 (PMC8627665; doi:10.1099/mgen.0.000310)
Supplement: Supplementary material 1 [file mgen-7-0310-s001.pdf]

Supplementary Material

Python Scripts - snp_align_gen.py

```
import argparse
import glob
import sys
import os


"""
MAKES SNP ALIGNMENT FROM SNIPPY OUTPUTS (*.vcf + *.aligned.fa)
"""

def parseargs():
    parser =
argparse.ArgumentParser(formatter_class=argparse.ArgumentDefaultsHelpFormat
ter)
    parser.add_argument("input", help="input path, can be folder of vcfs or
wildcard path")
    parser.add_argument("maskedgenomes", help="folder. must contain one
masked genome per vcf ending in .aligned.fa")
    parser.add_argument("output", help="file name for output fasta
alignment")
    parser.add_argument("-p","--percentmissing", help="percentage of SNP
positions allowed to be missing before strain is removed",default=20)
    parser.add_argument("--useref", help="include reference in alignment",
action='store_true')
    parser.add_argument("--nonsnps", help="include reference in alignment",
action='store_true')

    args = parser.parse_args()
    return args


def collect_SNPs(filepaths):
    strains = []
    snps = {}
    refpos = {}
    for file in filepaths:
        name = os.path.basename(file).replace(".vcf","")
        strains.append(name)
        f = open(file,"r").read().splitlines()
        for line in f:
            if line[0] != "#" and "TYPE=snp" in line:
                col = line.split("\t")
                pos = col[1]
                ref = col[3]
                mut = col[4]
```

```
                if pos not in snps:
                    snps[pos] = {(ref,mut):[name]}
                    refpos[pos] = ref
                else:
                    if (ref,mut) in snps[pos]:
                        snps[pos][(ref,mut)].append(name)
                    else:
                        snps[pos][(ref,mut)] = [name]
    # print(snps)
    return snps,strains,refpos

def get_genomes(strainls,folderpath):
    if folderpath[-1] != "/":
        folderpath += "/"
    genomedict = {}
    for i in strainls:
        ingenome = open(folderpath+i+".aligned.fa","r").read().splitlines()
        genomeseq = "".join(ingenome[1:])
        # print(genomeseq[:10],len(genomeseq))
        genomedict[i] = genomeseq
    return genomedict

def get_genome_call(pos,strain,genomedict):
    genome = genomedict[strain]
    call = genome[pos-1]
    return call

def make_alignment(snpdict,genomedict,strains,args,refpos):
    outalign = {}
    if args.useref:
        outalign["Reference"] = ""
    for strain in strains:
        outalign[strain] = ""
    for pos in sorted(map(int,list(snpdict.keys()))):
        for strain in strains:
            toadd = ""
            for mut in snpdict[str(pos)]:
                # print(i,mut,len(snpdict[str(pos)][mut]))
                if strain in snpdict[str(pos)][mut]:
                    toadd = mut[1]
            if toadd == "":
                call = get_genome_call(pos,strain,genomedict)
                toadd = call
            outalign[strain]+=toadd
        if args.useref:
            outalign["Reference"] += refpos[str(pos)]


    return outalign
```

```python
def filter_alignments(outalign,perc):
    fails = []
    for strain,align in outalign.items():
        c = 0
        for n in align:
            if n in ["N","n","X","x","-"]:
                c+=1
        missingperc = float(c)/len(align)
        if missingperc*100 > float(perc):
            fails.append(strain)
    return fails


def main():
    args = parseargs()

    inp = args.input
    filels = []
    if "*" in inp:
        filels = glob.glob(inp)
    else:
        if inp[-1] == "/":
            filels = glob.glob(inp+"*.vcf")
        else:
            filels = glob.glob(inp + "/*.vcf")

    if len(filels) == 0:
        sys.exit("input path found no vcf files")

    snpdict,strains,refpos = collect_SNPs(filels)

    genomedict = get_genomes(strains,args.maskedgenomes)

    outalign = make_alignment(snpdict,genomedict,strains,args,refpos)
    # print(len(refpos.values()))

    fail = filter_alignments(outalign,args.percentmissing)

    outf = open(args.output,"w")
    outf1 = open(args.output + "failed_calls.txt", "w")
    for i in outalign:
        if i not in fail:
            outf.write(">{}\n{}\n".format(i,outalign[i]))
        else:
            outf1.write(i+"\n")

    outf.close()
    outf1.close()
```

```
if __name__ == '__main__':
    main()
```
